# Dysregulated tryptophan metabolism: driving T cell subsets and PI3K-Akt pathway alterations in Hashimoto’s thyroiditis

**DOI:** 10.3389/fimmu.2025.1605739

**Published:** 2025-09-02

**Authors:** Lijian Zhang, Xinrui Zhou, Tingwei Cheng, Qiong Wang, Xiaoyan Pei, Lei Yu, Guoxi Jin

**Affiliations:** Department of Endocrinology, The First Affiliated Hospital of Bengbu Medical University, Bengbu, Anhui, China

**Keywords:** tryptophan metabolism, Hashimoto’s thyroiditis, T cell subsets, PI3K-Akt signaling pathway, immune

## Abstract

**Purpose:**

This study explored the role of tryptophan (Trp) metabolism in Hashimoto’s thyroiditis (HT) pathogenesis using clinical samples and animal models, given the unclear mechanisms and limited treatments of HT.

**Methods:**

Clinically, serum Trp, lactic acid, and alanine levels in 10 HT patients and 10 healthy controls were measured by ELISA. In animal experiments, female C57BL/6 mice were divided into Con, HT, HT+T (Trp supplemented), and HT+I (Trp metabolism inhibitor IDO1/TDO-IN-4 treated) groups. After inducing autoimmune thyroiditis, various tests were conducted, including ELISA for inflammation factors, HE staining for thyroid pathology, flow cytometry for T cell subsets, RNA-seq for gene expression, Western Blotting for PI3K-Akt pathway proteins, and CIBERSORT for immune cell analysis.

**Results:**

HT patients had significantly lower serum Trp levels. The HT group showed thyroid damage and increased inflammation factors. Trp supplementation alleviated thyroid damage and reduced inflammation factors, while the inhibitor worsened them. Trp also regulated T cell subsets and immune cell environment. RNA-seq and Western Blotting indicated Trp’s impact on immune response and PI3K-Akt pathway.

**Conclusion:**

Trp metabolism abnormality is associated with HT. Trp supplementation can alleviate HT progression by regulating T cell function and the PI3K-Akt pathway, while inhibiting Trp metabolism exacerbates it. This suggests Trp metabolism’s potential as a therapeutic target for HT.

## Highlights

This study demonstrates significantly decreased serum Trp levels in HT patients, suggesting an association between Trp metabolic abnormalities and HT pathogenesis. By showing Trp’s impact on inflammation, T cell subsets, and the PI3K-Akt pathway in animal models, it uncovers a novel mechanistic link. These findings highlight Trp supplementation as a potential therapeutic strategy to mitigate HT progression, offering a new avenue for managing this common endocrine disorder. The mechanistic insights into Trp’s immunomodulatory effects advance understanding of HT and may inform future treatments targeting metabolic pathways in autoimmune endocrine diseases.

## 1 Introduction

The treatment of HT currently relies primarily on levothyroxine replacement therapy. However, even after thyroid function is controlled, patients may still experience persistent extrathyroidal symptoms, such as constipation, diarrhea, edema, anxiety, and hair loss (1, 2). Although studies have confirmed that both cellular and humoral immunity are involved in HT, the specific mechanisms remain to be further clarified. A comprehensive approach using diverse research methods is needed to conduct in-depth exploration and analysis of the immune mechanisms of HT (3).

To explore the potential impact of amino acid metabolism abnormalities in HT, we used metabolomics to analyze serum samples from HT patients and healthy individuals. We identified Trp, lactic acid, and alanine as differential metabolites for further study. Trp, an essential amino acid, is metabolized through three main pathways: the kynurenine (Kyn) pathway, the 5-hydroxytryptamine (5-HT) pathway, and the indole-3-pyruvate (I3P) pathway (4–6). The Kyn pathway, accounting for 95% of Trp metabolism, occurs in immune and epithelial cells and is initiated by Indoleamine 2,3-dioxygenase (IDO) or Tryptophan 2,3-dioxygenase (TDO) (6). In this metabolic pathway, IDO1 initiates the conversion of Trp to Kyn, which acts as an Aryl Hydrocarbon Receptor (AhR) ligand; activated AhR translocates to the nucleus and up-regulates IDO1 in dendritic cells (DCs), establishing an IDO1–Kyn–AhR positive-feedback loop. This loop confers DCs with an immunosuppressive phenotype that drives regulatory T cells (Tregs) differentiation, suppresses effector T cells, and maintains self-tolerance (5, 7). A Study reports that HT patients exhibit fewer IDO^+^ plasmacytoid dendritic cells (pDCs), elevated serum Trp, a reduced Kyn/Trp ratio, and heightened *in vitro* IFN-α responses (8). These data implicate Trp-metabolic imbalance in HT pathogenesis via immune-homeostasis disruption; dissecting this mechanism may inform new diagnostics and therapeutics.

In HT patients, the helper T cells (Th)/Tregs balance is markedly skewed toward Th1, Th2, and Th17 subsets (9, 10). Within CD4^+^ T cells, AhR is absent in Th1 and Th2, high in Th17, and low in Tregs (11). The Kyn pathway bidirectionally regulates the Th17/Tregs balance via AhR-dependent mechanisms, inhibiting Th17 polarization and promoting Tregs differentiation (12–14), positioning it as a potential therapeutic target in HT.

Collectively, Trp metabolism appears to modulate the balance of Th-cell subsets and thereby participate in the pathogenesis of HT. Although the precise mechanisms operating in HT remain incompletely elucidated, significant advances in understanding Trp metabolism have already been achieved in other disorders—including COVID-19, glioma, and inflammation-induced depression—where it has demonstrated promising clinical translational potential (6). Consequently, investigating the interplay between Trp metabolism and HT is scientifically imperative and may open new avenues for immune-metabolic reprogramming–based targeted therapies.

## 2 Materials and methods

### 2.1 Patient population

Ten female HT patients and ten healthy women (25–65 years) were recruited from the Endocrinology Outpatient Clinic of the First Affiliated Hospital of Bengbu Medical University between June and December 2023.

Inclusion criteria:

- HT patients: ① Positive serum TPOAb (>60 IU/mL) or TgAb (>60 IU/mL); ② Thyroid ultrasound revealing heterogeneous parenchymal texture, with or without hypoechoic areas or thyroid nodules.
- Control group: ① Normal thyroid function and thyroid autoantibodies; ② No thyroid diseases or autoimmune diseases; ③ Absence of infectious disease and normal hepatic, renal, glycemic, and lipid profiles.

Exclusion criteria:

- Use of medications affecting thyroid function or presence of other thyroid disorders;
- Coexisting autoimmune disease or malignancy;
- current treatment with immunosuppressive or glucocorticoid agents;
- active infection, inflammation, recent trauma, or other causes.

After confirming eligibility, we obtained written informed consent and collected peripheral blood for serum isolation. Demographic and clinical data were extracted from medical records, anonymized, and stored under secure identifiers to safeguard participant privacy.

### 2.2 Experimental animals

Female C57BL/6 mice (6–8 weeks old) were purchased from GemPharmatech Co., Ltd. (Nanjing, China).

### 2.3 Reagents and instruments

Main reagents and instruments are in **Supplementary Materials**.

### 2.4 Animal model

The establishment of the HT mice model in this study was performed as described in the reference (PMID: 34124070) (15).

Female C57BL/6 mice (6–8 weeks old) were used to establish the model. After 1-week adaptive feeding, they were randomly divided into 4 groups (n=10 per group):

- Control (Con) group: From the second week onward, the mice received 0.05% NaCl as drinking water. On the same week, they were given multiple subcutaneous injections of PBS at the dorsal, abdominal, and cervical regions, and daily intraperitoneal PBS injections were initiated and continued until the end of the experiment. Two weeks later, multi-site subcutaneous PBS injections were repeated.
- HT model (HT) group: Beginning in the second week, the mice received 0.05% NaI as drinking water and were administered the first series of subcutaneous injections of porcine thyroglobulin (200 μg per mouse) emulsified in complete Freund’s adjuvant (CFA) at multiple sites on the dorsal, abdominal, and cervical regions. Concurrently, daily intraperitoneal injections of PBS were initiated and maintained until the end of the experiment. Two weeks later, a second set of subcutaneous injections of porcine thyroglobulin (200 μg per mouse) emulsified in incomplete Freund’s adjuvant (IFA) was given at multiple sites.
- HT + Trp (HT+T) group: The regimen was identical to that of the HT group, except that the daily intraperitoneal injection was replaced with 20 mg/kg Trp (dissolved in PBS).
- HT + Kyn-pathway inhibitor (HT+I) group: The regimen was identical to that of the HT group, except that the daily intraperitoneal PBS injection was replaced with 20 mg/kg of the Kyn-pathway inhibitor (IDO1/TDO-IN-4) administered every other day. IDO1/TDO-IN-4 is a potent dual inhibitor of both IDO1 and TDO and therefore functions as an effective Kyn-pathway inhibitor (16). A stock solution of 20 mg/ml was prepared in DMSO and diluted ten-fold with 20% (w/v) SBE-β-CD in normal saline immediately before use.

Four weeks after the final immunization challenge, the mice were euthanized, and serum, thyroid glands, and spleens were collected for subsequent analyses.

### 2.5 Experimental assays

#### 2.5.1 ELISA

Serum concentrations of Trp, lactic acid, and alanine in human samples were quantified with commercially available kits. Mouse serum levels of TPOAb, TgAb, IL-17, and Trp were likewise determined using standardized ELISA kits.

#### 2.5.2 HE staining

Thyroid specimens were fixed in 4% paraformaldehyde, processed through graded ethanol, embedded in paraffin, sectioned at 4 μm, and stained with hematoxylin and eosin for histopathological evaluation.

#### 2.5.3 Flow cytometry

Splenic lymphocytes were isolated and surface-stained for CD4 and CD25, followed by intracellular staining for IL-4, IFN-γ, and IL-17A using fluorochrome-conjugated antibodies. Data were acquired on a flow cytometer and analyzed to quantify the expression profiles of the indicated markers.

#### 2.5.4 RNA-seq

Total RNA sequencing was performed by Biomarker Technologies Co., Ltd. (Beijing, China). RNA quality assessment, library construction, library quality control, and Illumina sequencing were executed according to the company’s standardized pipelines. Raw reads were uploaded to the BMKCloud platform (www.biocloud.net) for adapter/quality trimming, alignment to the reference genome, transcript quantification, and differential expression analysis, ensuring a comprehensive and reproducible data interpretation.

#### 2.5.5 Western blot

Frozen thyroid tissues stored at −80°C were lysed in RIPA buffer containing protease and phosphatase inhibitors. Protein concentrations were determined by BCA assay, and 30 µg of total protein per sample was separated by SDS-PAGE and transferred to PVDF membranes. After blocking, membranes were probed overnight at 4°C with primary antibodies against mTOR, p-mTOR (Ser2448), PI3K, p-PI3K (Tyr607), Akt, and p-Akt (Ser473). GAPDH was used as the loading control. HRP-conjugated secondary antibodies were applied, and signals were detected by enhanced chemiluminescence. Band intensities were quantified to assess the effect of Trp metabolism on PI3K-Akt pathway activity.

### 2.6 Immune cell landscape analysis

Immune-cell composition in tissue samples was determined with the CIBERSORT algorithm implemented in R. The signature gene matrix (LM22) was obtained from the original publication (PMID 25822800) (17). The CIBERSORT R package was installed and run to extract immune cell proportion data and perform visualization. The Wilcoxon rank-sum test was used to compare the immune cell proportions under different conditions. P<0.05 was considered statistically significant. After running the CIBERSORT R package, estimated immune-cell proportions were extracted and visualized. Between-group differences were evaluated by the Wilcoxon rank-sum test; P < 0.05 was considered statistically significant.

### 2.7 Statistics analysis

All statistical analyses were performed with SPSS 21.0 (IBM Corp., Armonk, NY, USA). Normally distributed data are expressed as mean ± standard deviation (M ± SD) or mean ± standard error of the mean (M ± SEM); differences between two groups were evaluated by two-sample t-test, and those among multiple groups by one-way or multi-way ANOVA followed by appropriate *post-hoc* tests. Non-normally distributed data are presented as median and interquartile range [Mdn (Q1, Q3)] and analyzed with the Mann-Whitney U or Wilcoxon rank-sum test. Statistical significance was set at a two-tailed P < 0.05.

## 3 Result

### 3.1 Alterations of serum metabolites in patients with HT

**Figure 1:**
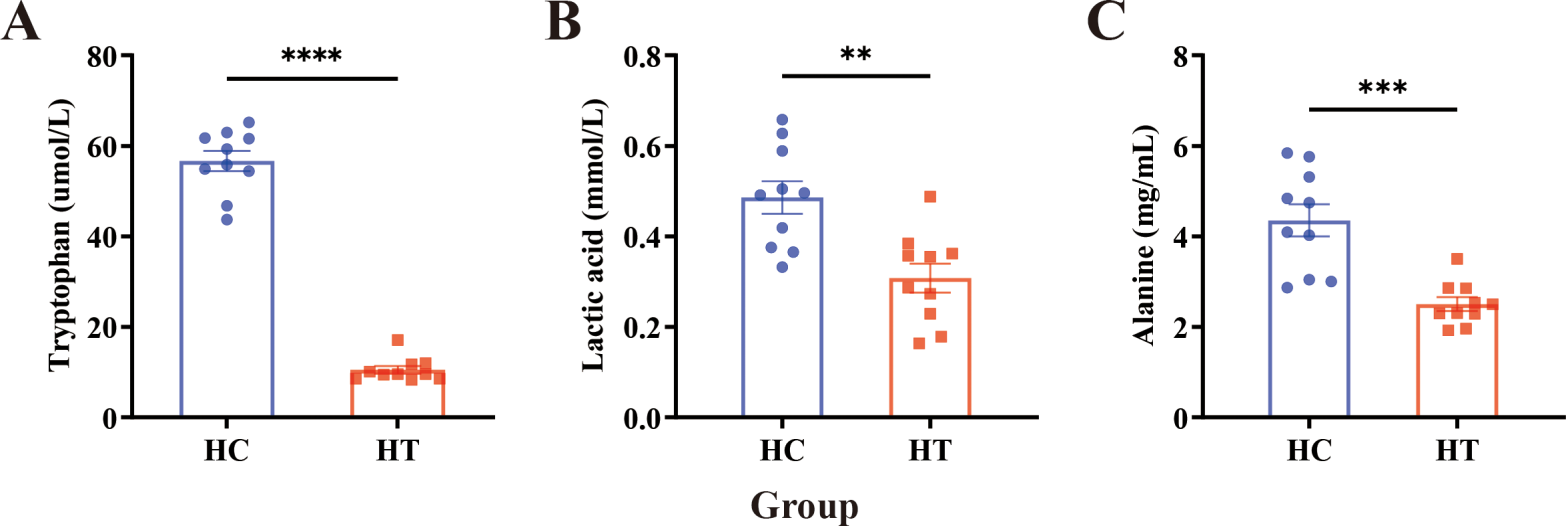
ELISA detection of serum metabolite levels in HT patients (HT) and healthy individuals (HC). **(A–C)** Serum concentrations of **(A)** tryptophan, **(B)** lactate, and **(C)** alanine in healthy controls and HT patients. Data are expressed as mean ± SEM; n = 10 per group. Statistical comparisons were performed using an unpaired two-tailed Student’s t-test. Asterisks denote significant differences: **P<0.01, ***P<0.001, ****P<0.0001.

The baseline demographic and clinical characteristics of the enrolled participants are summarized in **Table 1**. Serum samples from both groups were analyzed using the ELISA method, and the results are presented in **Figure 1**. ELISA quantification revealed significantly lower concentrations of serum Trp (P < 0.0001), lactate (P < 0.01), and alanine (P < 0.001) in the HT group compared with the healthy controls (HC group), with Trp exhibiting the greatest decrease (**Figure 1**).

**Table 1:** Clinical characteristics of patients with hashimoto’s thyroiditis (HT group) and healthy individuals (HC group).

Clinical variable	HC (n=10)	HT (n=10)	*P*
Age (year)	45.00 ± 9.83	49.60 ± 9.97	0.313
TT3 (nmol/L)	1.73 ± 0.35	2.55 ± 4.01	0.532
TT4 (nmol/L)	98.37 ± 11.57	66.41 ± 30.07	0.009*
FT3 (pmol/L)	5.09 ± 0.37	4.20 ± 0.87	0.008*
FT4 (pmol/L)	15.70 ± 1.47	12.66 ± 3.56	0.023*
TPOAb (U/mL)	47.90 (41.08, 55.75)	> 1300.00 (548.2, > 1300.00)	< 0.001*
TgAb (U/mL)	15.90 (<15.00, 19.30)	235.30 (93.48, > 500.00)	0.002*
TSH (mIU/L)	2.92 (2.14, 4.34)	4.66 (2.62, 34.02)	0.162
urine iodine (ug/L)	135.70 ± 71.66	198 ± 105.01	0.139
Duration of disease (year)	–	6.65 (0, 11.33)	–
Medication status			
- Stable levothyroxine ≥ 6 wk	–	6 (60%)	–
- Treatment-naïve newly diagnosed	–	3 (30%)	–
- ≥4-wk wash-out	–	1 (10%)	–

Normally distributed data are expressed as mean ± SD, and intergroup comparisons were performed using Student’s t-test. Non-normally distributed data are presented as Mdn (Q1, Q3), and the Mann-Whitney U test was employed for intergroup comparisons, *indicating P<0.05.

### 3.2 Impact of Trp on inflammation in HT mice

**Figure 2:**
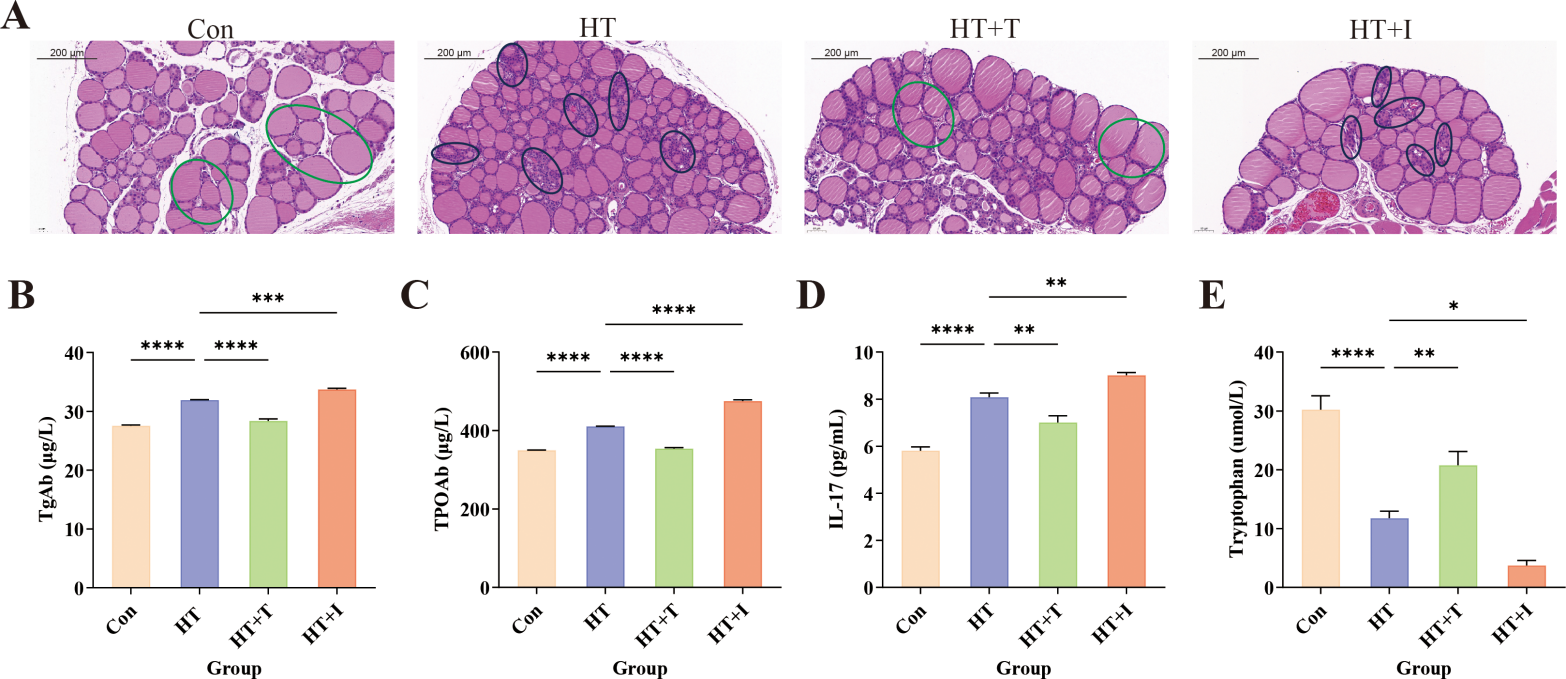
Effects of Trp on HT mice. **(A)** Representative HE-stained thyroid sections (original magnification, ×20) showing follicular architecture and lymphocytic infiltration; representative areas of inflammatory cell infiltration in the HT and HT+I groups are circled in black, while representative healthy regions in the Con and HT+T groups are circled in green. Three independent mice per group were analyzed. **(B–E)** Serum levels of **(B)** thyroglobulin antibody (TgAb), **(C)** thyroid peroxidase antibody (TPOAb), **(D)** interleukin-17 (IL-17), and **(E)** Trp determined by ELISA. For ELISA, three biologically independent samples were assayed in triplicate; data are presented as mean ± SEM. Statistical comparisons were performed using two-way ANOVA. Asterisks denote significant differences: *P<0.05, **P<0.01, ***P<0.001, ****P<0.0001.

Following model induction, thyroid tissues were collected and subjected to HE staining to observe the morphological structure of thyroid follicular cells and lymphocyte infiltration (**Figure 2A**). In the Con group, follicular cells were neatly arranged, follicles were round or oval, and staining was light red, with no inflammatory cell infiltration. In contrast, the HT group exhibited extensive follicular destruction, marked atrophy or loss of follicles, and dense perifollicular lymphocytic infiltrates. Trp supplementation (HT+T group) partially preserved follicular integrity and reduced inflammatory infiltrates compared with HT group. Conversely, blockade of the Kyn pathway (HT+I group) exacerbated follicular atrophy and amplified lymphocytic infiltration beyond the severity observed in the HT group.

Serum levels of TgAb (**Figure 2B**), TPOAb (**Figure 2C**), and IL-17 (**Figure 2D**) were quantified by ELISA. Relative to Con group, HT group exhibited marked elevations of TgAb, TPOAb, and IL-17 (all P < 0.0001). Compared with the HT group, the levels of TgAb (P<0.0001), TPOAb (P<0.0001), and IL-17 (P<0.01) in the HT+T group were significantly decreased, while the levels of TgAb (P<0.001), TPOAb (P<0.0001), and IL-17 (P<0.01) in the HT+I group were significantly increased. These findings indicate that Trp mitigates HT-associated inflammation, whereas blockade of the Kyn pathway aggravates it, implicating this pathway in the suppression of thyroid autoimmunity.

Furthermore, the Trp levels in serum were quantified by ELISA (**Figure 2E**). Relative to the Con group, the Trp levels in the HT group were significantly decreased (P<0.0001). Compared with the HT group, the Trp levels in the HT+T group were significantly increased (P<0.01), and the Trp levels in the HT+I group were significantly decreased (P<0.05).

### 3.3 Impact of Trp on CD4^+^ T cell subsets in HT mice

**Figure 3:**
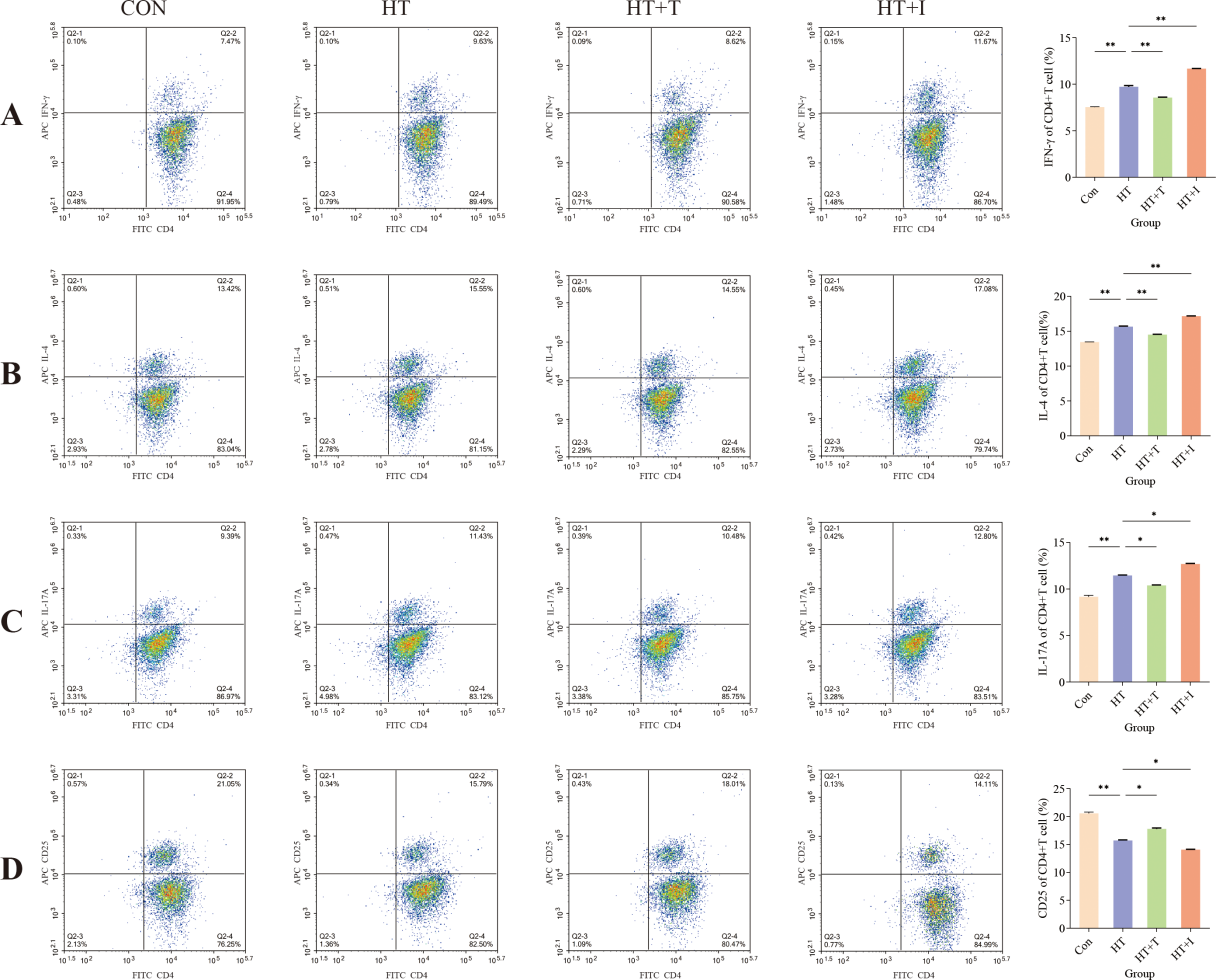
Flow cytometric analysis of splenic T cell subsets. **(A)** Percentage of IFN-γ^+^CD4^+^ (Th1) cells. **(B)** Percentage of IL-4^+^CD4^+^ (Th2) cells. **(C)** Percentage of IL-17A^+^CD4^+^ (Th17) cells. **(D)** Percentage of CD25^+^CD4^+^ cells. For each group, splenocytes from three biologically independent mice were stained and analyzed in triplicate. Data are presented as mean ± SEM. Comparisons among groups were performed using two-way ANOVA. Asterisks denote significant differences: * P<0.0001, ** P<0.00001.

The proportion of CD4^+^ T cell subpopulations in mouse spleens was measured using flow cytometry to explore the impact of Trp on immune cell function. Relative to the Con group, the proportions of Th1 (**Figure 3A**), Th2 (**Figure 3B**), and Th17 (**Figure 3C**) cells in the HT group were significantly increased (all P < 0.00001), while the proportion of CD4^+^CD25^+^ cells (**Figure 3D**) was significantly decreased (P < 0.00001). Compared with the HT group, Trp administration (HT+T group) reversed these changes, the proportions of Th1 (P < 0.00001), Th2 (P < 0.00001), and Th17 (P < 0.0001) cells were significantly decreased, and the proportion of CD4+CD25+ cells was significantly increased (P < 0.0001). In contrast, the proportions of Th1 (P < 0.00001), Th2 (P < 0.00001), and Th17 (P < 0.0001) in the HT+I group were significantly increased, and the proportion of CD4+CD25+ cells was significantly decreased (P < 0.0001). Although CD25 is also expressed by activated conventional T cells, CD4^+^CD25^+^ cells are highly enriched for Foxp3^+^ Tregs and can therefore serve as a surrogate Treg population in the absence of intracellular Foxp3 staining (18).

### 3.4 Impact of Trp on the transcriptome of thyroid tissue in HT mice

**Figure 4:**
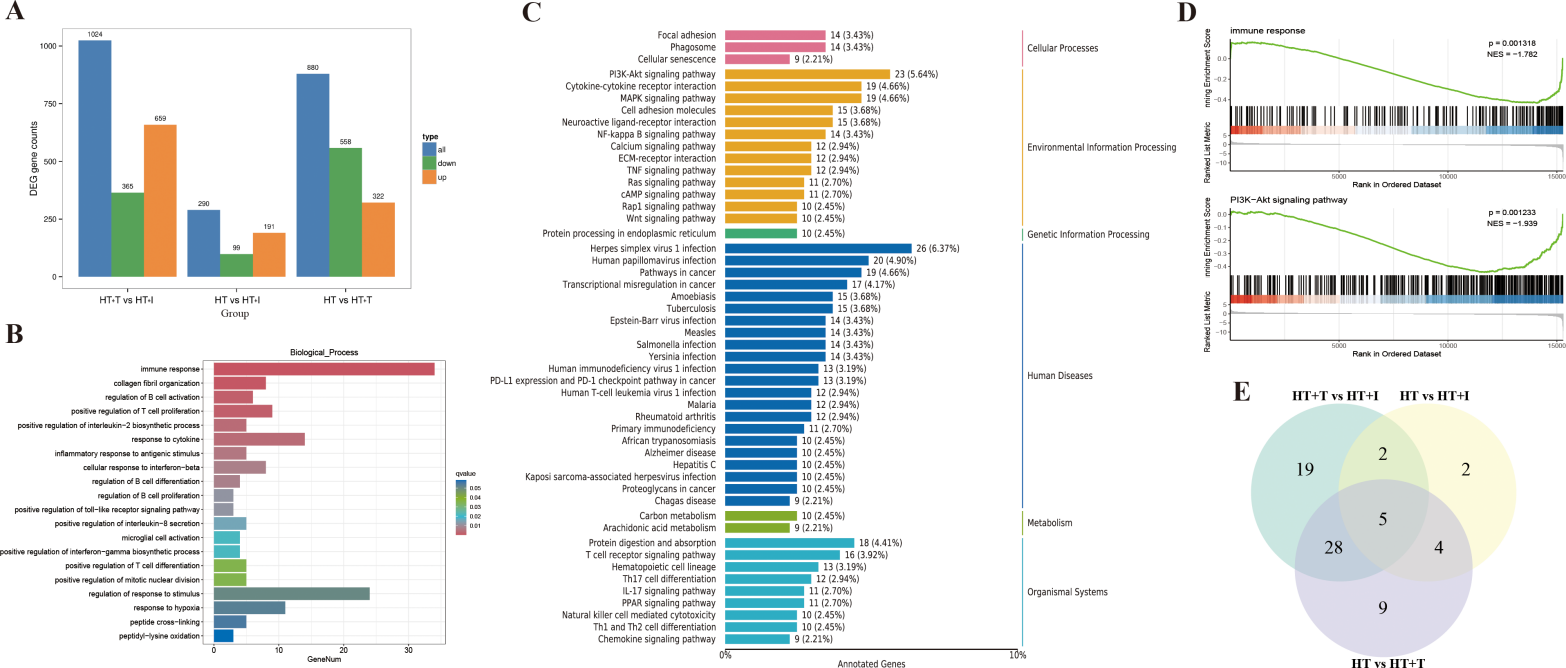
Transcriptional analysis of thyroid tissue from HT mice. Each group (HT, HT+T and HT+I) comprised three biologically independent samples. **(A)** Bar chart of DEGs. **(B)** GO biological process enrichment bar chart of DEGs between HT and HT+T groups. **(C)** KEGG pathway classification plot of DEGs between HT and HT+T groups. **(D)** GSEA analysis plot between HT and HT+T groups. **(E)** Venn diagram of intersecting genes.

To systematically evaluate the influence of Trp supplementation on the thyroid transcriptome of HT mice, bulk RNA-sequencing was conducted. Differential expression analysis (|log_2_ fold change| ≥ 2 and FDR < 0.05) revealed that Trp supplementation profoundly remodeled the thyroid transcriptome (**Figure 4A**). Compared with the HT group, the HT+T group exhibited 880 differentially expressed genes (DEGs) (322 up-regulated, 558 down-regulated) and the HT+I group exhibited 290 DEGs (191 up-regulated, 99 down-regulated). In the comparison between HT+T and HT+I groups, the HT+I group displayed 1,024 DEGs relative to HT+T group (659 up-regulated, 365 down-regulated).

Gene Ontology (GO) enrichment analysis (**Figure 4B**) revealed significant differences in gene expression between the HT and HT+T groups, particularly in immune response, where more than 30 genes were significantly enriched (q-value<0.01). Kyoto Encyclopedia of Genes and Genomes (KEGG) pathway analysis (**Figure 4C**) showed significant enrichment of the PI3K-Akt signaling pathway in both groups, with 23 DEGs involved (5.64% of total DEGs). Consistently, Gene Set Enrichment Analysis (GSEA) (**Figure 4D**) confirmed significant enrichment of immune response (NES = -1.782, P = 0.0013) and the PI3K–Akt signaling pathway (NES = -1.939, P = 0.0012).

Transcriptomic data were subjected to KEGG functional annotation, and pathways containing ≥10 DEGs were retained for comparative analysis. The intersection of such pathways across treatment groups is depicted in **Figure 4E**. The most prominently represented categories included hematopoietic cell lineage, Yersinia infection, Epstein-Barr virus infection, the Ras signaling pathway, and the PI3K-Akt signaling pathway, each exhibiting substantial enrichment of DEGs. Because the PI3K-Akt signaling pathway is pivotal in orchestrating immune cell maturation, differentiation, trafficking, and survival (19), it was selected for subsequent mechanistic investigation and experimental validation.

### 3.5 Effects of Trp the PI3K–Akt signaling pathway in HT mice

**Figure 5:**
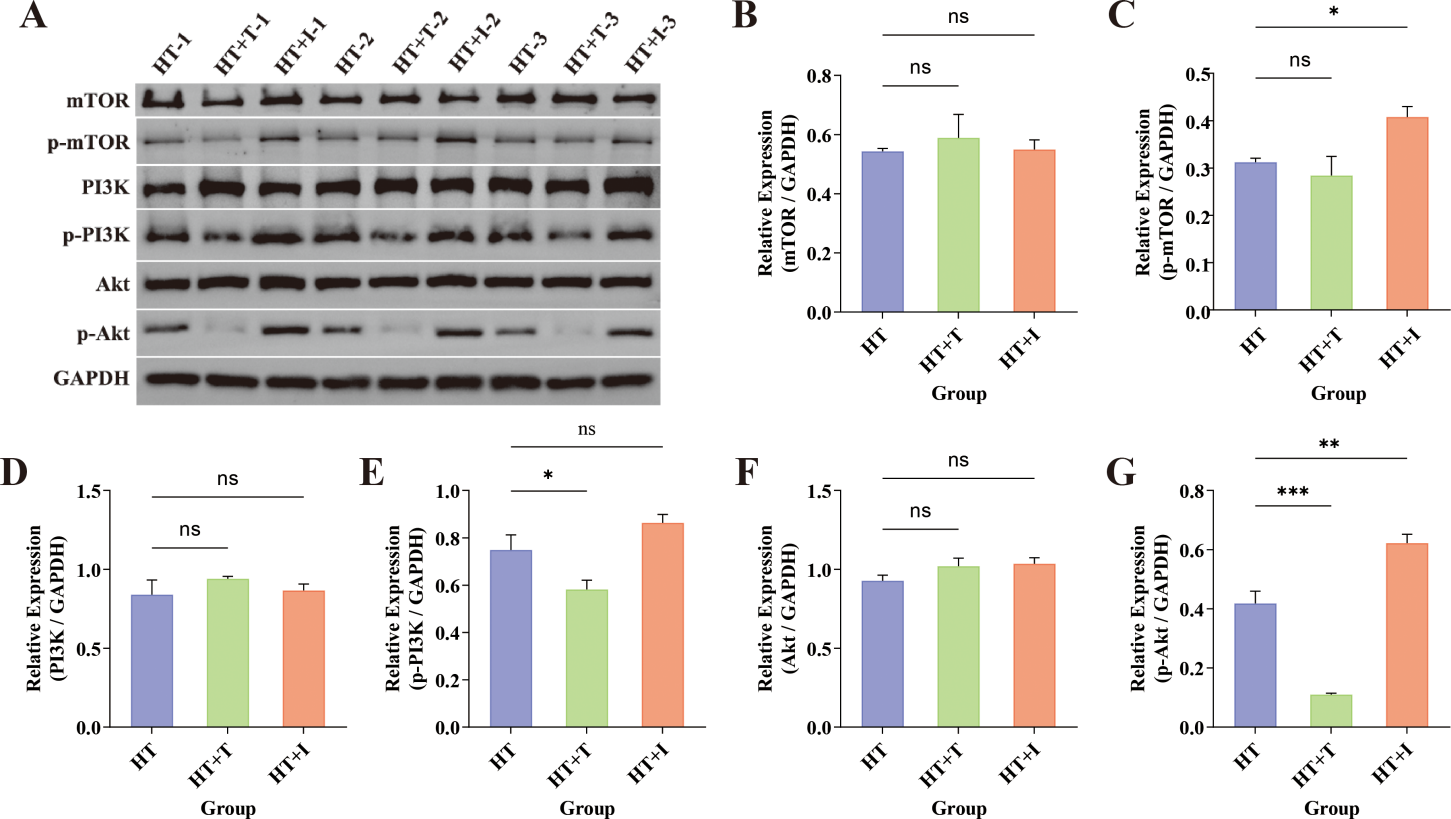
Western Blot analysis of thyroid tissue from HT mice. **(A)** Representative immunoblots for mTOR (~250–289 kDa), p-mTOR (~289 kDa), PI3K (~126 kDa), p-PI3K (~80 kDa), Akt (~60 kDa), and p-Akt (~60 kDa) across the three experimental groups (n = 3 biologically independent samples per group). GAPDH served as the loading control. **(B–G)** Quantification of relative protein expression normalized to GAPDH for **(B)** mTOR, **(C)** p-mTOR, **(D)** PI3K, **(E)** p-PI3K, **(F)** Akt, and **(G)** p-Akt. Data are presented as mean ± SEM. Inter-group comparisons were performed using one-way ANOVA. ns, not significant; *P<0.05; **P<0.01; ***P<0.001.

To determine whether Trp influences HT through the PI3K-Akt axis, the thyroid levels of key pathway proteins (mTOR, p-mTOR, PI3K, p-PI3K, Akt, and p-Akt) were quantified by western blotting (**Figure 5A**). Densitometric analysis with ImageJ revealed that total mTOR (**Figure 5B**), PI3K (**Figure 5D**), and Akt (**Figure 5F**) remained unchanged in both HT+T and HT+I groups relative to HT group (P > 0.05). In contrast, the expression levels of p-PI3K (**Figure 5E**) and p-Akt (**Figure 5G**) proteins were decreased in the HT+T group (P<0.05 and P<0.001, respectively), while there was no significant difference in the expression of p-mTOR (**Figure 5C**) protein (P>0.05). Conversely, the expression levels of p-mTOR (P<0.05) and p-Akt (P<0.01) proteins were increased in the HT+I group, and there was no significant difference in the expression of p-PI3K protein (P>0.05).

### 3.6 Trp modulates thyroidal immune cell infiltration in HT mice

**Figure 6:**
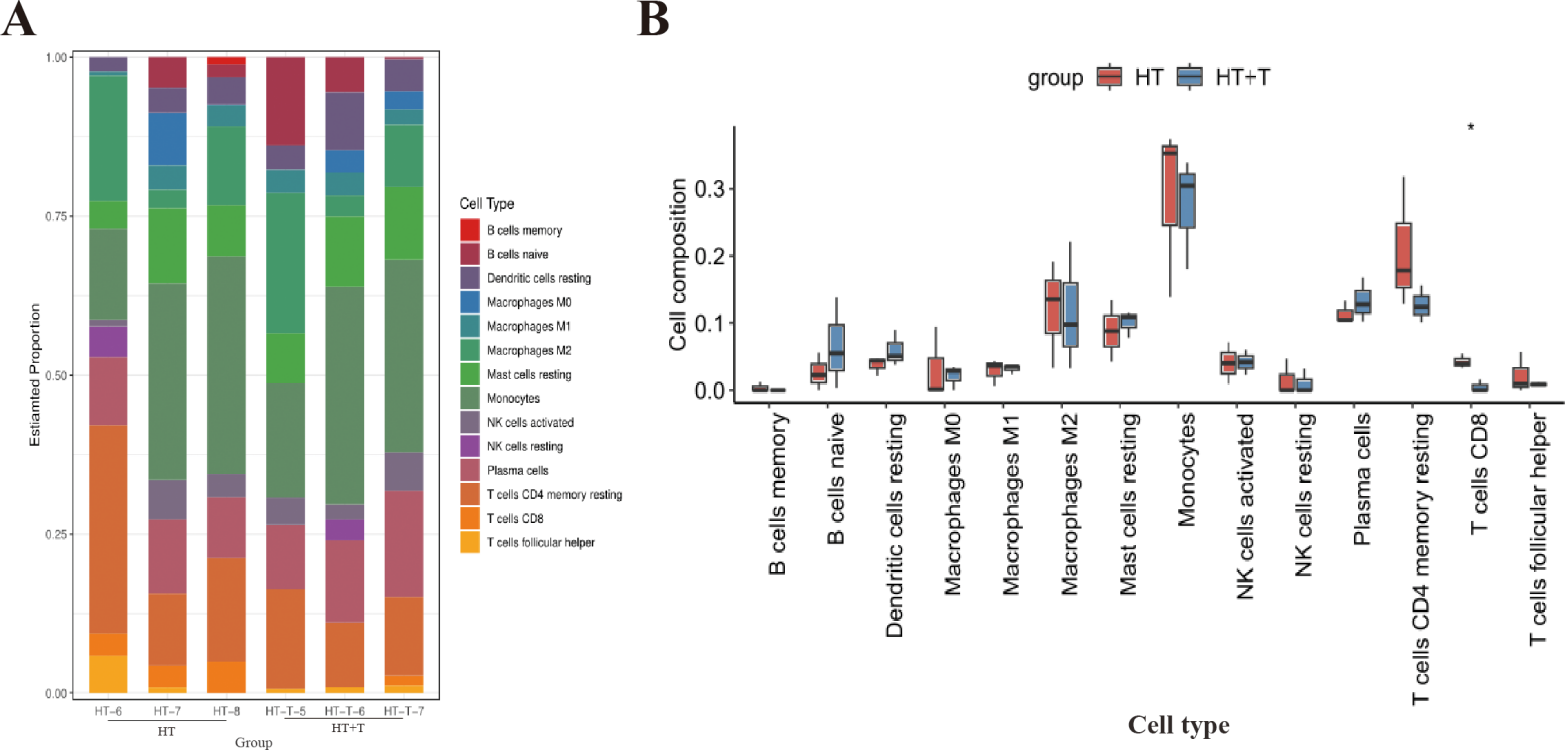
Immune cell landscape in thyroid tissue of HT versus HT+T mice. **(A)** Relative abundances of 22 immune cell subsets estimated by CIBERSORT based on bulk RNA-seq profiles (n = 3 per group). **(B)** Comparative analysis of immune cell proportions between HT and HT+T groups. Data are shown as box-and-whisker plots (median ± interquartile range). Statistical significance was determined by the two-tailed Wilcoxon rank-sum test; *P<0.05.

To explore the regulatory effect of Trp on immune cells in the thyroid of HT mice, the CIBERSORT algorithm was used to analyze the RNA-seq dataset of thyroid tissue to characterize the composition of immune cell subpopulations (**Figure 6A**). Comparison of immune cell distribution between the HT and HT+T groups revealed significant differences (**Figure 6B**). Relative to HT group, HT + T group significantly reduced the abundance of CD8^+^ T cells (P < 0.05) and, to a lesser extent, resting memory CD4^+^ T cells (P < 0.05), with CD8^+^ T cells exhibiting the greatest fold change.

## 4 Discussion

### 4.1 The relationship between HT and Trp metabolism

Trp catabolism is increasingly recognized as a pivotal contributor to autoimmune pathogenesis. A convergent finding across multiple studies is an elevated serum Kyn/Trp ratio in patients with diverse autoimmune diseases (ADs) and in corresponding animal models, a parameter that correlates with disease activity and supports its utility as a biomarker (20–25). Building on these observations, our study further investigated the changes in serum metabolite levels in HT patients through clinical sample analysis. Consistent with previous reports, circulating Trp concentrations were markedly reduced in HT patients (**Figure 1A**). In the HT animal model, we found that Trp supplementation could alleviate the progression of HT, while treatment with IDO1/TDO-IN-4 worsened it (**Figure 2**). Collectively, these data implicate dysregulated Trp metabolism in HT pathogenesis and establish modulation of Trp availability as a rational therapeutic strategy for this disorder.

**Figure 2E** shows that, relative to the HT group, administration of IDO1/TDO-IN-4 produced a paradoxical decrease in serum Trp. In theory, inhibition of the rate-limiting enzymes IDO1 and TDO should reduce Trp flux through the Kyn pathway and thereby elevate circulating Trp. The unexpected decline can be reconciled by two non-mutually exclusive explanations: (i) the inhibitor failed to achieve an effective concentration or activity *in vivo*, or (ii) the blocked Trp was diverted to alternative Trp metabolic branches rather than the Kyn pathway. A recent study in a murine depression model supports the latter possibility. The TDO inhibitor paeoniflorin increased hepatic 5-HT/Trp ratios while decreasing Kyn/Trp ratios, indicating that TDO blockade diverts Trp away from the Kyn pathway and toward 5-HT synthesis (26). This unexpected finding indicates that the *in vivo* effects of IDO/TDO inhibition on the global Trp metabolic network are more intricate than current theoretical frameworks suggest, and a comprehensive elucidation of the underlying molecular mechanisms and metabolic fluxes is urgently warranted in future investigations.

### 4.2 Effects of Trp metabolism on T cell subsets

Lymphocytic infiltration constitutes the hallmark histopathological feature of HT (27). Nevertheless, the precise mechanisms underlying its action in HT remain to be fully elucidated. In HT, the infiltrate is composed predominantly of T lymphocytes, whose sustained targeting of thyroid follicular cells results in parenchymal destruction, progressive fibrosis, and, ultimately, glandular atrophy with attendant hypothyroidism (28). Given the immunoregulatory potency of Trp metabolites (29, 30), we hypothesized that altered Trp catabolism may mechanistically link metabolic dysregulation to immune cell-mediated thyroid injury.

Trp degradation products critically modulate immune cell function. DCs, as key antigen-presenting cells, can modulate the activation and differentiation of various Th subsets by sensing environmental signals (31). In an inflammatory setting, increased IDO1 expression in DCs promotes Tregs differentiation, suppresses effector T cell activity, and enhances the Kyn pathway, which breaks down Trp into Kyn (32). Kyn binds to and activates the AhR, a transcription factor involved in regulating immune cell differentiation and function. AhR activation up-regulates its own expression and increases IDO1 expression in DCs, forming a positive feedback loop (IDO1-Kyn-AhR) that enhances the immunosuppressive capacity of DCs (33, 34). Trp metabolism also regulates immune responses through AhR-independent pathways. The breakdown of Trp can influence the metabolism of immune cells by activating GCN2 kinase and other pathways. This indirectly regulates the activity of the mTOR pathway, thereby limiting T cell proliferation (30). Notably, this metabolic-immune crosstalk may simultaneously reshape the distribution of immune cells in both the peripheral circulation and the target tissue.

Previous study utilizing transcription-factor profiling of peripheral-blood mononuclear cells (PBMCs) have demonstrated that the homeostatic balance among Th1/Tregs, Th2/Tregs, and Th17/Tregs subsets is disrupted in HT patients, manifesting as a predominance of Th1, Th2, and Th17 cell populations (9). Meta-analyses of newly diagnosed autoimmune thyroiditis cohorts and corresponding animal models further converge on an elevated Th17/Tregs ratio as a cardinal immunological signature of the disease (35–38). Consistent with these observations, flow-cytometric quantification of splenic CD4^+^ T cell subsets in our murine model revealed that Trp partially restored the Th/Tregs balance (**Figure 3**). Collectively, these suggest that Trp may improve the immune microenvironment by modulating peripheral CD4^+^ T cell subset homeostasis.

To characterize the immune landscape of thyroid tissue, we applied the CIBERSORT algorithm, which deconvolves bulk RNA-seq data into the relative abundance of 22 distinct immune cell subsets and has become a widely accepted tool for quantifying tissue-infiltrating leukocytes in immune-mediated diseases (17). CIBERSORT analysis demonstrated that Trp markedly decreased the relative abundance of CD8^+^ T cells and resting memory CD4^+^ T cells in the thyroid of HT mice, with CD8^+^ T cells showing the most pronounced decrease (**Figure 6**).

CD8^+^ T cells are potent drivers of autoimmunity; they inflict tissue injury through direct cytotoxicity and pro-inflammatory cytokine release (39). In HT, the activation and expansion of CD8^+^ T cells can exacerbate the immune response, triggering an attack on thyroid tissue (3). Moreover, both TPOAb and TgAb antigens are recognized by CD8^+^ T cells and participate in thyroid tissue destruction (40). With respect to the interplay between tryptophan metabolism and CD8^+^ T cells, studies have demonstrated that tryptophan insufficiency activates GCN2 kinase, which in turn suppresses mTORC1 signaling. This triggers an energetic reprogramming of CD8^+^ T cells—most notably a marked reduction in glycolysis—that ultimately impedes their clonal expansion and acquisition of effector functions (41). The results suggest that Trp could contribute to the pathogenesis of HT through its regulation of CD8^+^ T cell infiltration and function within the thyroid. Overall, Trp may attenuate HT progression via a dual mechanism: rebalancing peripheral T cell subsets while simultaneously restraining tissue-infiltrating lymphocytes.

### 4.3 Trp metabolism and the PI3K-Akt signaling pathway

HT, a prevalent autoimmune endocrine disorder, involves aberrant regulation of multiple signaling pathways (42, 43). Here, transcriptomic profiling of murine thyroid tissue showed that Trp exerts its therapeutic effects in HT chiefly by modulating immune-related biological processes (**Figure 4B**). According to the KEGG pathway enrichment analysis, the PI3K-Akt signaling pathway showed significant enrichment in the environmental information processing category (**Figure 4C**).

During T cell development, the PI3K-Akt signaling pathway is implicated in the β-selection checkpoint, facilitating the progression of T cells from CD4^−^CD8^−^ to CD4^+^CD8^+^ by supporting cell survival, proliferation, and metabolic processes (44). Inhibitors of this pathway can selectively suppress the activation and proliferation of Tregs while having a relatively smaller impact on other CD4^+^ T cells (45). Studies have shown that blocking PI3Kγ or PI3Kδ in CD8^+^ T cells alone can enhance their anti-tumor properties (46). The PI3K-Akt signaling pathway is also strongly linked to the development of thyroid diseases. Existing studies have shown that long-term iodine deficiency or excessive iodine intake may contribute to the onset and progression of HT by altering the DNA methylation levels of the PRKAA2 and ITGA6 genes within the PI3K-Akt signaling pathway (47). Similarly, Trp and its metabolites can inhibit inflammation through the PI3K-Akt signaling pathway (48).

Western blot analysis revealed no significant inter-group differences in total protein levels of mTOR, PI3K, or Akt following treatment with Trp or IDO1/TDO-IN-4 (**Figure 5**). In contrast, phosphorylation status was markedly altered. Relative to the HT group, phosphorylation of PI3K and Akt was significantly attenuated in the HT+T group, whereas phosphorylation of mTOR and Akt was markedly elevated in the HT+I group. These data indicate that Trp suppresses PI3K-Akt signaling via diminished PI3K and Akt phosphorylation, whereas IDO1/TDO-IN-4 activates the same pathway through increased mTOR and Akt phosphorylation.

Emerging evidence indicates that the Kyn-AhR axis exerts bidirectional control over Akt phosphorylation. In a murine model of autoimmune hepatitis, hepatic IDO1 activation accelerates the Kyn pathway, which via AhR signaling attenuates Th17 differentiation, expands Tregs, and suppresses Akt phosphorylation, ultimately ameliorating liver injury (49). These findings align with our observation that Trp-mediated IDO1 activation dampens PI3K-Akt signaling in HT. Conversely, in glioma, elevated TDO2-derived Kyn engages AhR to potentiate PI3K-Akt signaling and accelerate malignant cell proliferation (50, 51). This divergence likely stems from two factors. First, the experimental strategies differ: the former study enhanced Kyn production by activating IDO1, whereas the latter abolished Kyn synthesis by deleting TDO2; our approach employed the dual IDO1/TDO inhibitor IDO1/TDO-IN-4, which simultaneously targets both branches of the Kyn pathway. Second, cell-intrinsic differences dictate distinct AhR-downstream signaling networks, yielding opposing effects on Akt activity. Future studies should therefore utilize selective IDO1 or TDO2 inhibitors to dissect cell-type-specific modulation of the Akt pathway and to elucidate the underlying mechanistic distinctions.

### 4.4 Limitations and future directions

In summary, Trp metabolism may influence T cells via the PI3K-Akt signaling pathway and thereby participate in the initiation and progression of HT. These findings not only provide a new perspective on HT pathogenesis but also offer important clues for future immunomodulatory therapeutic strategies targeting HT.

Nevertheless, several limitations must be acknowledged. First, the limited sample size restricts the generalizability and reliability of the results. Second, the molecular intermediates that link specific Trp catabolites to discrete T cell subsets remain to be delineated. Third, the observation that serum Trp levels increase in the HT mouse model after treatment with IDO1/TDO-IN-4 remains incompletely explained, and the underlying mechanism requires further clarification. Finally, although altered Akt phosphorylation was documented, the causality of this event was not confirmed through pharmacological or genetic Akt blockade.

Future investigations should therefore (i) validate the immunomodulatory effects of Trp in larger, multi-center HT cohorts; (ii) integrate isotope-tracing metabolomics, single-cell transcriptomics, and conditional knockout models to dissect the Trp–immune cell regulatory circuitry; and (iii) employ selective Akt inhibitors to establish direct functional links. These efforts will be critical for translating mechanistic insights into therapeutic strategies that improve clinical outcomes and quality of life for patients with HT and related thyroid disorders.

## Data Availability

The data presented in the study are deposited in the NCBI SRA repository, accession number PRJNA1304611.
