# Supplementary material for: Dysregulated tryptophan metabolism: driving T cell subsets and PI3K-Akt pathway alterations in Hashimoto’s thyroiditis
Source: Front Immunol. 2025 Sep 2;16:1605739. doi: 10.3389/fimmu.2025.1605739 (PMC12436296; doi:10.3389/fimmu.2025.1605739)
Supplement: Supplementary file 1 [file Table1.docx]

**Main Reagents for Animal Modeling**

| Reagent | Manufacturer | Catalog Number |
| --- | --- | --- |
| PBS | Solarbio | P1003 |
| Complete Freund's Adjuvant | Beyotime | P2036-50ml |
| Porcine Thyroglobulin (PTG) | Wuhan Curity Bio-Tech Co., Ltd | CD-Z-1013L |
| Sodium Iodide | Shanghai Yuanye Biotechnology Co., Ltd. | S30233 |
| IDO1/TDO-IN-4 | MedChemExpress | HY-151108 |

**ELISA Detection Reagents and Instruments**

| Reagent or Instrument | Manufacturer |  | Catalog Number |
| --- | --- | --- | --- |
| Human Alanine ELISA Detection Kit | Shanghai Enlink Bio-Tech Co., Ltd |  | YJ493043 |
| Human Lactic Acid ELISA Detection Kit | Shanghai Enlink Bio-Tech Co., Ltd |  | YJ694033 |
| Tryptophan Enzyme-Linked Immunosorbent Assay (ELISA) Kit | Shanghai Zeyue Biotechnology Co., Ltd |  | ZY-Trp-serum |
| Mouse TPO-Ab ELISA Detection Kit | Shanghai Enlink Bio-Tech Co., Ltd |  | YJ7390420 |
| Mouse TGAB ELISA Detection Kit | Shanghai Enlink Bio-Tech Co., Ltd |  | YJ749503 |
| Microplate Reader | Tecan |  | - |

**Main Reagents and Instruments for HE Staining**

| **Reagent or Instrument** | **Manufacturer** | **Catalog Number or Model** |
| --- | --- | --- |
| Anhydrous Ethanol | Chemical Reagents Co., Ltd, Sinopharm Group | 100092683 |
| Xylene | Chemical Reagents Co., Ltd, Sinopharm Group | 10023418 |
| HE Staining Kit | Servicebio | G1003 |
| Neutral Gum | Chemical Reagents Co., Ltd, Sinopharm Group | 10004160 |
| Dehydrator | DIAPATH | Donatello |
| Embedding Machine | Wuhan Junjie Electronic Co., Ltd | JB-P5 |
| Microtome | Shanghai Leica Instruments Co., Ltd | RM2016 |
| Frozen Sectioning Machine | Wuhan Junjie Electronic Co., Ltd | JB-L5 |
| Tissue Spreader | Kedi Instruments Equipment Co., Ltd, Jinhua City, Zhejiang Province | KD-P |
| Staining Machine | DIAPATH | Giotto |
| Oven | Tianjin Laibo Rui Instruments Equipment Co., Ltd | GFL-230 |
| Upright Optical Microscope | Nikon, Japan | Nikon Eclipse E100 |
| Imaging System | Nikon, Japan | Nikon DS-U3 |

**Main Reagents and Instruments for Flow Cytometry**

| Reagent or Instrument | Manufacturer | Catalog Number or Model |
| --- | --- | --- |
| Ficoll Cell Separation Solution | Sigma | 17544602 |
| PBS | Gibco | C10010500BT |
| Mouse BD Fc Block | BD | 553141 |
| FITC anti-mouse CD4 Antibody | Biolegend | 100405 |
| APC anti-mouse CD25 Antibody | Biolegend | 113708 |
| APC anti-mouse IL-4 Antibody | Biolegend | 504105 |
| APC anti-mouse IFN-γ Antibody | Biolegend | 505809 |
| APC anti-mouse IL-17A Antibody | Biolegend | 506915 |
| Mouse BD Fc Block | BD | 553141 |
| BD Cytofix/Cytoperm™ Plus Fixation/Permeabilization Solution Kit with BD GolgiPlug™ | BD | 555028 |
| Anti-mouse CD3 | Absin | S0B0661 |
| Anti-mouse CD28 | Absin | S0B0003E |
| Biosafety Cabinet | Thermo | 1300-A2 |
| Fluorescence Microscope | Mshot | MF53 |
| Flow Cytometer | NovoCyte | 2060R |
| CO₂ Incubator | Thermo | 3111 |

**Main Reagents and Instruments for Western Blotting**

| Reagent or Instrument | Manufacturer | Catalog Number |
| --- | --- | --- |
| p-mTOR (Ser2448) | Proteintech | 67778-1-Ig |
| mTOR | Proteintech | 66888-1-Ig |
| PI3K | Abcam | ab302958 |
| p-PI3K p85 alpha (Tyr607) | Affinity | AF3241 |
| AKT | CST | #9272 |
| p-AKT (Ser473) | CST | #4060 |
| GAPDH | Proteintech | 60004-1-Ig |
| Goat anti- Rabbit IgG-HRP | Absin | abs20040 |
| Goat anti-Mouse IgG-HRP | Absin | abs20039 |
| HT-Mini04 Mini Vertical Electrophoresis Tank | Hongji | - |
| HT-ZY02 Mini Transfer Electrophoresis Tank | Hongji | - |
| HT-600C Universal Electrophoresis Power Supply | Hongji | - |
| DYY-6C Dual Stable-Time Electrophoresis Power Supply | Beijing Liuyi | - |
| SH-Advance523 Multi-Functional Ultra-Sensitive Imaging System | Shenhua | - |
